# Simultaneous identification and determination of flavonoids in *Dendrobium officinale*

**DOI:** 10.1186/s13065-018-0403-8

**Published:** 2018-04-12

**Authors:** Chunhua Zhou, Zhenshan Xie, Zhouxi Lei, Yuechun Huang, Gang Wei

**Affiliations:** 1 School of Pharmaceutical Sciences, Guangzhou University of Chinese Medicine, 232 Outer Circle Road East, Guangzhou Higher Education Mega Center, Guangzhou, 510006 China; 2 The First Affiliated Hospital, Guangzhou University of Chinese Medicine, Guangzhou, 510405 Guangdong People’s Republic of China

**Keywords:** *Dendrobium officinale*, Different origins, Flavonoid glucosides, Identification, Quantitative analysis

## Abstract

**Background:**

The quality of material medicine resources has had a considerable impact on the development of the health industry, which has created a bottleneck for traditional Chinese medicine (TCM). *Dendrobium officinale*, which has been widely used for health prevention in TCM, has become a high-nutritive health food that is strongly recommended by many white-collar workers and people who pay more attention to their health. The aim of this study was to develop a method to authenticate and evaluate *D. officinale* from different origins via simultaneous qualitative and quantitative analyses of flavonoid glycosides. Ultra-high-performance liquid chromatography-electrospray ionization/mass spectrometry was used for the structural elucidation of the compounds.

**Results:**

9 characteristic peaks, including those representing 7 flavonoid C-glycosides and 2 flavonoid *O*-glycosides, were identified. Additionally, the contents of 5 representative flavonoid glucosides in 25 batches of *D. officinale* from different sources were determined. To further investigate the different sources of the 25 batch samples, principal component analysis (PCA) and hierarchical cluster analysis (HCA) were carried out. A study on the methodology revealed that all results were reliable.

**Conclusions:**

This method is an efficient tool for the rapid identification of the different geographical origins of *D. officinale* and provides references for the quality evaluation of other natural products. 
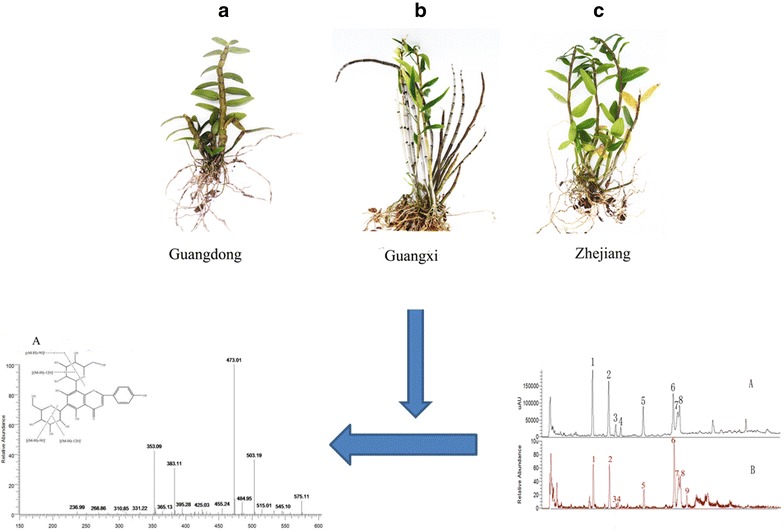

## Introduction

The Dendrobium genus is one of the largest genera of Orchidaceae [1]. There are thousands of species of Dendrobium all over the world [2]. Dozens of species, including *Dendrobium officinale*, *Dendrobium nobile*, *Dendrobium huoshanense* and *Dendrobium chrysanthum*, are grown in China [3]. Among all of these Dendrobium species, *Dendrobium officinale* is one of the most popular for its functions in TCM, such as tonifying the stomach, promoting fluid, nourishing yin and clearing heat [4, 5]. The fresh stem of *D. officinale* can be orally consumed directly, and it can also be used as a soup stock or tea. Meanwhile, modern pharmacology studies have indicated that *D. officinale* has some beneficial bioactivities, such as anti-oxidant, anti-tumor, hypoglycemic, and hypoglycemic activities and gastrointestinal regulatory functions [6–8].

**Fig. 1:**
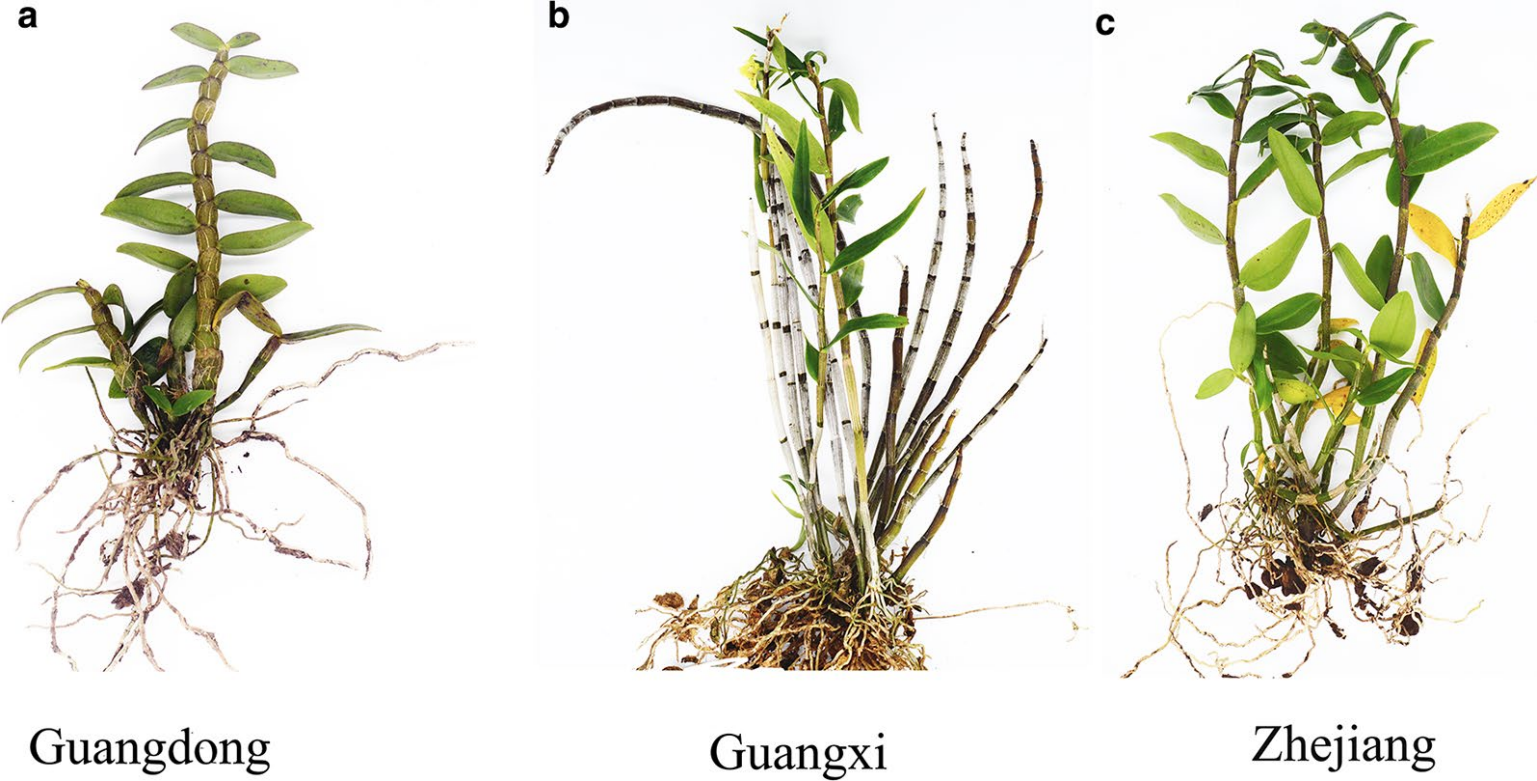
The pictures of the medical plant, *Dendrobium officinale.* **a** Collected from Danxia landform area (Guangdong), **b** collected from Guangnan area (Guangxi), **c** collected from Zhejiang province

Generally, the quality of genuine regional remedies are outstanding [9]. *D. officinale* from the Danxia landform region has become a genuine medicinal material since the Northern and Southern Dynasties of China, which were approximately 1500 years ago. Since then, the herbal medicine *D. officinale* has mainly been distributed in some Danxia landform regions located in the Fujian and Guangdong provinces, and the Danxia landform area was the first main habitat of *D. officinale*. However, since the Song Dynasty, the Guangnan area (consisting of the Yunnan and Guangxi provinces) and Zhejiang province became the two main habitats of this herb. After considerable consultation of ancient herbal documents and on-the-spot investigation, we discovered that *D. officinale* that grew in the abovementioned 3 habitats were different from each other in character and shape (as shown in Fig. 1). Thus, we assumed that the content and types of chemical compounds in this herb may be different. The main active ingredients of *D. officinale* are phenols and polysaccharides [10–13]. To date, studies on *D. officinale* have mainly focused on the polysaccharides [14–16]. However, polysaccharides are ubiquitous in Dendrobium species. Flavonoids are a widespread group of phytochemicals with diverse biological functions and significant substances in plants that not only play a key role in the pharmaceutical industry but also serve as excellent chemical markers for quality control of medicinal plants [17–19]. Several reports have studied the flavonoids in different parts of *D. officinale* [20]. However, no reports have been published on the effects of the producing region on the flavonoids in *D. officinale*, and this is not comprehensive to study quality control. By comparing the constituents of *D. officinale* from the three main genuine producing regions and by searching for common specific components, the chemical differences in the different producing regions were revealed. This was critical for the synthetic evaluation of *D. officinale*.

To establish a comprehensive evaluation system for *D. officinale*, we utilized UHPLC-ESI–MS/MS fingerprint chromatography. Then, the chemical markers were identified, and the contents of 25 batch samples were collected from the Danxia landform region in Zhejiang province and tested. Furthermore, principal component analysis (PCA) and hierarchical cluster analysis (HCA) were utilized to analyze the different sources of *D. officinale*. These results showed that this method could be successfully used for identifying specific discriminating markers to identify *D. officinale* from different geographical environments and to improve the quality evaluation system of *D. officinale.*

## Experimental

### Chemicals and reagents

Apigenin-6,8-di-C-β-d-glucoside, isoviolanthin and apigenin-6-C-β-d-xyloside-8-C-β-d-Glucoside were isolated from the leaves of *D. officinale* via preparative liquid chromatography as reference substances for the experiments. Rutin, naringin and schaftoside were obtained from the National Institute Control of Pharmaceutical and Biological Products (Guangzhou, China). The purity of all compounds mentioned above was over 96%, and the compounds were of HPLC grade; their chemical structures were identified by comparing their UV, IR, ESI/MS, and NMR spectra with other published reports.

HPLC grade methanol was purchased from Merck (Darmstadt, Germany). Ultrapure water was prepared using a Milli-Q water purification system (MA, USA). Analytical grade methanol and ammonium acetate (CH_3_CO_2_NH_4_) were obtained from Damao Chemical Corporation, Tianjin, China. Tetrahydrofuran was purchased from Mreda (USA).

Twenty-five samples of fresh, mature *D. officinale* stems were collected from different regions of China in the Danxia landform area (Fujian, Guangdong, and Jiangxi), Guangnan area (Guangxi and Yunnan) and Zhejiang province. Of these, 3 batches were from Guangdong province (No. GD1–GD3), 3 batches were from Jiangxi province (No. JX1–JX3), 1 batch was from Fujian province (No. FJ), 8 batches were from Zhejiang province (No. ZJ1–ZJ8), 5 batches were from Yunnan province (No. YN–YN5), and 5 batches were from Guangxi province (No. GX1–GX5).

### Preparation of standard solutions and sample preparation

A set of standard solutions were prepared by appropriate dilution of the stock solution with methanol. They were then diluted to construct different calibration plots in the following ranges: 9.25–1850 ng/mL for apigenin-6,8-di-C-β-d-glucoside, 8.43–1686 ng/mL for apigenin-6-C-β-d-xyloside-8-C-β-d-glucoside, 7.14–1428 ng/mL for schaftoside, 16.5–3300 ng/mL for rutin, and 120–2390 ng/mL for isoviolanthin.

The samples were dried and ground into powder; 1.0 g powder was placed in 100 mL Erlenmeyer flasks and ultrasonically extracted twice for 45 min each with 50 mL methanol. The total extract was concentrated and evaporated to dryness, and the residue was re-dissolved with an adequate amount of methanol: water (80:20, v/v). Then, the solution was transferred to a 2 mL volumetric flask and diluted to a constant volume (10 mL). Prior to injection, all solutions were filtered through a 0.22 μm microporous membrane. All the solutions were stored in a refrigerator at 4 °C before analysis.

### Qualitative and quantitative analysis

Quantitative analyses were performed using a UHPLC system equipped with a vacuum degasser, quaternary pump, auto-sampler and ultraviolet detector (Thermo Separation Products Inc., Riviera Beach FL, USA). All data were processed on a Finnigan Xcalibur 2.0 advanced chromatography workstation (Thermo Quest Corporation, San Jose, CA, USA). The studies were conducted on a Hypersil GOLD C18 (100 × 2.1 mm ID, 1.9 μm, Thermo, USA) with a suitable guard column (C18, ODS, 1.9 μm, 4.0 × 3.0 mm). The mobile phase consisted of methanol (A) and a 10 mM (v/v) ammonium acetate aqueous solution (B) with a linear gradient elution at a flow rate of 200 μL/min. The elution program was carried out according to the following profile: 0–10 min, 20–23% A; 10–15 min, 23–26% A; 15–16 min, 26–30% A; 16–25 min, 30–35% A; 25–30 min, 35–42% A; 30–35 min, 42–35% A. The column temperature was maintained at 30 °C, and the sample injection volume was 3 μL. The detection wavelength was 340 nm. MS analysis was performed on a Thermo Finnigan LCQ FLEET equipped with an ion trap mass spectrometer with an electrospray ionization interface and an ultraviolet detector. Nitrogen was used as the sheath and auxiliary gas, and helium was used as the collision gas. The ESI/MS spectra were acquired in both positive and negative ion modes. The ESI source conditions were as follows: spray voltage of 2800 V in negative ion ESI mode, 3500 V in positive ion ESI mode, capillary temperature of 350 °C, sheath gas flow rate of 30 (arbitrary units), auxiliary gas flow rate of 10 (arbitrary units), and scan range for both MS and MS/MS between *m/z* 150 and 1000.

Quantitative analyses were run on an Agilent 1100 system. Chromatographic separations were carried out on a Kromasil 100-5 C18 (250 × 4.6 mm, 5.0 µm) maintained at 30 °C. The mobile phases consisted of water containing tetrahydrofuran: acetonitrile: methanol (10:22:5) (A) and 0.05% phosphoric acid (B), and the elution gradient was set as follows: 0–10 min, 10–11% A; 10–25 min, 11–11.5% A; 25–32 min, 11.5–12% A; 32–42 min, 12–12.5% A; 42–52 min, 12.5–13.5% A; 52–75 min, 13.5–14% A. The flow rate was 1 mL/min, and the detection wavelength was 340 nm.

### Method validation

The 5 standard solutions were diluted to six different concentrations with methanol to investigate the linearity. The concentration of the standard solutions was represented on the X-axis (X), the chromatographic peak area was on the Y-axis (Y), and the results were analyzed by linear regression statistics. The repeatability was evaluated by carrying out six replicate analyses of the same sample (YN4). The RSD_S_ for the retention time and peak area were calculated as measures of repeatability.

The precision was investigated by analyzing the sample on the same day (intra-day) and between 3 consecutive days (inter-day). For precision absorption of the sample solution of *D. officinale* from Yunnan (YN4), the samples were analyzed 6 times continuously, and the RSD_S_ for the retention time and peak area were calculated to evaluate the intra-day and inter-day precision. To evaluate the stability of the sample, selected sample (YN4) was analyzed at room temperature at 0, 2, 4, 6, 8, 12, and 24 h after preparation, and the stability was expressed by the RSD_S_ for the retention time and peak area.

The recoveries of the 5 compounds were determined by spiking the sample (YN4) with suitable amounts (approximately 100% of the contents) of the standard compounds that were previously determined. The actual amounts in relation to the theoretically present amounts were expressed as a percentage of the recovery.

### Chemometric analysis

To distinguish the relatively homogeneous groups of the 25 *D. officinale* from different origins, the HCA multivariate analysis technique was performed using SPSS software (SPSS 23.0 for Windows, SPSS Inc., USA).

## Results and discussion

### Characterization of flavonoid glycosides

**Fig. 2:**
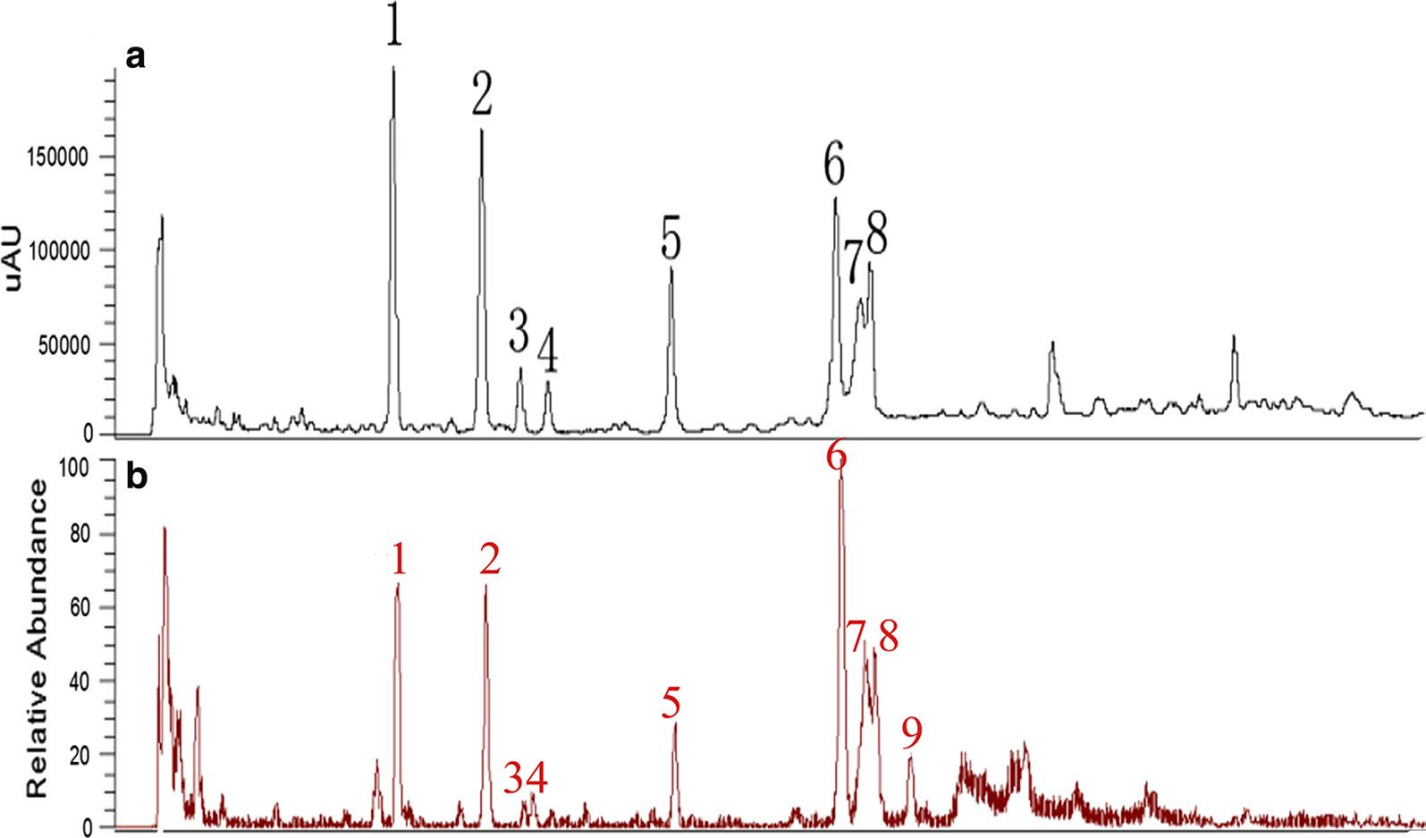
UHPLC-UV chromatograms of *Dendrobium officinale* (**a**), The UHPLC-ESI/MS (TIC) fingerprint of *Dendrobium officinale* (**b**)

UHPLC-ESI–MS/MS was adopted to characterize the target constituents of *D. officinale*. Both positive and negative ion modes were utilized to ionize the flavonoid glycosides, and negative mode ESI was found to be sensitive for flavone glycosides of *D. officinale*, which showed the [M-H]^−^ deprotonated ions in the negative mode ESI–MS spectra for all the flavonoid glycoside ingredients. The product ion scans of the 9 flavonoid glycosides re shown in Fig. 2. By comparing the retention times from the UV and ESI-MS^n^ spectra with literature data, we identified and deduced the possible structures of the 9 main constituents, including flavonoid *O*-glycosides and flavonoid C-glycosides. The TIC chromatogram is shown in Fig. 2b. The retention times (t_R_), MS and MS^2^ spectral data and identification of the flavonoids are listed in Table 1. Some compounds were unambiguously identified by comparing them with the reference compound. However, because of the unavailability of authentic compounds, some of the peaks could only be tentatively assigned.

**Table 1:** MS date for characterization of compounds in *Dendrobium officinale* by UHPLC-ESI–MS/MS

Peak no	RT (min)	Negative ions (m/z)	MS^n^	Identification
1	7.45	593[M-H]^−^	MS^2^: 503.19, 473.01, 383.11, 353.09	Apigenin-6,8-di-C-β-d-glucoside
2	9.82	563[M-H]^−^	MS^2^: 503.08, 473.04, 443.08, 383.10, 353.07	Apigenin-6-C-β-d-xyloside-8-C-β-d-glucoside
3	10.85	563[M-H]^−^	MS^2^: 503.07, 473.03, 443.10, 383.06, 353.05	Isoschaftoside
4	11.58	563[M-H]^−^	MS^2^: 473.07, 443.10, 383.10, 353.09	Schaftoside
5	14.87	563[M-H]^−^	MS^2^: 473.09, 443.10, 383.18, 353.12	Apigenin-6-C-β-d-glucoside-8-C-β-d-xyloside
6	19.28	609[M-H]^−^	MS^2^: 301.04, 271.09; MS^3^: 271.07, 255.08, 179.00, 151.01	Rutin
7	20.04	577[M-H]^−^	MS^2^: 457, 383	Isoviolanthin
8	20.18	577[M-H]^−^	MS^2^: 457, 383	Violanthin
9	21.27	579[M-H]^−^	MS^2^: 417	Naringin

#### Characterization of flavonoid C-glycosides

In this study, 7 flavonoid C-glycosides were identified in *D. officinale* by peaks 1, 2, 3, 4, 5, 7 and 8, and the characteristic fragment patterns of flavonoid C-glycosides resulted from the cleavage of the glucosyl as follows: a series of fragment ions arising from the loss of [(M-H)-60]^−^, [(M-H)-90]^−^, [(M-H)-120]^−^, [(M-H)-90-120]^−^], [M-H-2 × 120]^−^, [(M-H)-120-CO]^−^, and [(M-H)-2 × 120-2CO]^−^ were the major fragmentation pathways in the MS or MS/MS. The flavonoid C-glycosides in *D. officinale* usually are present on the C-6 and C-8 position. Furthermore, glycosides on the C-6 position exhibited more fragmentation than those from the C-8 position. In the positive ESI/MS of *D. officinale*, the base peaks always appeared as [M + H]^+^ and [M + Na]^+^ ions, which were further fragmented by the successive losses of one molecule of H_2_O, leading to the product ions [M + H-18]^+^. The trials showed that the negative ion mode was more sensitive than the positive ion mode.

**Fig. 3:**
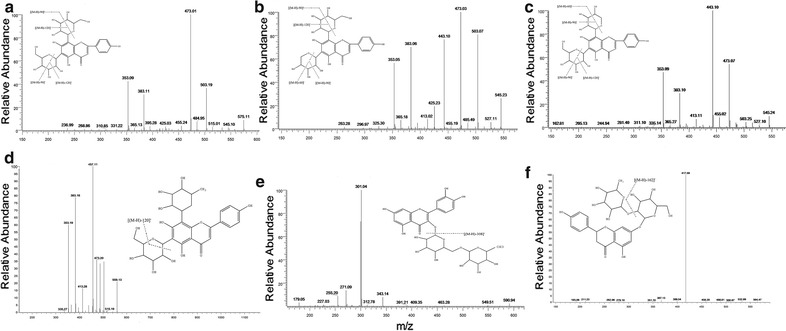
Chimical structures and MS/MS spectra of 6 flavonoids, Apigenin-6,8-di-C-β-glucoside (**a**), Isoschaftoside (**b**), Schaftoside (**c**), Violanthin (**d**), Rutin (**e**), Naringin (**f**)

Peak 1 represented [M-H]^–^at *m/z* 593. The fragment ion peaks shown in Fig. 3a, including 473 [(M-H)-120]^−^, 353 [(M-H)-120-90]^−^, were assigned to apigenin-6,8-di-C-β-d-glucoside by comparing with the standard. Peaks 2 and 3 represented a molecular ion [M-H]^−^ at *m/z* 563, which produced a similar MS^2^ base peak at *m/z* 473 [(M-H)-90]^−^ and an MS^3^ base peak at *m/z* 353 [(M-H-90-120]^–^. Upon comparison with the standards, peak 2 was established to be due to apigenin-6-C-β-d-xyloside-8-C-β-d-glucoside, whereas peak 3 was identified as isoschaftoside, as shown in Fig. 3b. Peaks 4 and 5 (in Fig. 3c) also represented the same molecular ion [M-H]^−^ at *m/z* 563, yielding product ions at *m/z* 443 [(M-H)-120]^−^ and 353 [(M-H)-120-90]^−^. Meanwhile, fragment ions at *m/z* 503 [(M-H)-60]^−^, *m/z* 383 [(M-H)-120-60]^−^ and *m/z* 353 [(M-H)-120-90]^−^ were also present. Moreover, these findings were consistent with literature data [21]. Compound 4 was unambiguously identified as schaftoside by comparing with the standard, and peak 5 was assigned to apigenin-6-C-β-d-glucoside-8-C-β-d-xyloside. Peaks 7 and 8 were identified as representing isoviolanthin and violanthin (Fig. 3d), which had similar molecular ions [M-H]^−^ at *m/z* 577 and the same fragment ions at 457 [(M-H)-120]^−^.

#### Characterization of flavonoid *O*-glycosides

The fragmentation behaviors of peak 6 with the loss of *m/z* 308, 146, 162, and 176 revealed the possible presence of flavonoid *O*-glycosides, whereas 308 Da was confirmed to be a typical fragment of rutinose. Furthermore, peak 6 molecular ions at 609.34 [M-H]^−^ and 610.82 [M + H]^+^, which produced fragments at *m/z* 301 [(M-H)-308]^−^, *m/z* 300 [(M-2H)-308]^−^, and *m/z* 303 in positive ion mode, were evidence of quercetin. Peak 6 was assigned to rutin, as shown in Fig. 3e, which was unambiguously identified via comparison with the reference standard. Peak 9 showed an [M-H]^−^ ion at *m/z* 579 and a fragment ion at *m/z* 417 due to the successive loss of 162 Da and was identified as naringin (shown in Fig. 3f).

### Method validation

As listed in Tables 2 and 3, all the calibration curves showed good linearity in their corresponding ranges for the 5 analyses (R^2^ > 0.999). The intra-day RSDs and inter-day RSDs of the 5 compounds were 0.50–2.57%, and 0.80–2.45%, respectively. The values for repeatability and stability were less than 2.60%, indicating that the sample possessed excellent stability over 24 h. The average recovery of the assay was between 100.72 and 102.11%, with RSDs of 0.93–2.93%. All the data indicated that the developed method is satisfactory for the qualitative and quantitative analysis of *D. officinale*.

**Table 2:** Regression equation, correlation coefficient (R^2^), linear range for 5 representative compounds from *Dendrobium officinale*

Compounds	Regression equation	R^2^	Linear range (ng/mL)
Apigenin-6,8-di-C-β-d-glucoside	y = 1770.70x + 11.105	0.9997	9.25–1850
Apigenin-6-C-β-d-xyloside-8-C-β-d-glucoside	y = 1889.51x + 5.476	0.9998	8.43–1686
Schaftoside	y = 2046.54x + 14.891	0.9998	7.14–1428
Isoviolanthin	y = 1719.51x − 4.4021	0.9999	119.51–2390
Rutin	y = 1202.72x + 11.916	0.9998	16.50–3300

**Table 3:** Repeatability, intraday and interday precisions, and stability, recovery of 5 representative compounds from *Dendrobium officinale*

Compounds	Repeatability RSD (%) (n = 6)	Precisions		Stability RSD (%) (n = 6)	Recovery (%)				
		Intra-day (n = 6)	Inter-day (n = 3)		Content (μg)	Spiked (μg)	Found (μg)	mean ± SD	RSD (%)
Apigenin-6,8-di-C-β-d-glucoside	0.61	0.50	0.80	0.43	21.62	22.21	44.62	101.54	1.24
Apigenin-6-C-β-d-xyloside-8-C-β-d-glucoside	1.10	1.31	1.28	1.31	14.51	15.20	29.93	101.04	0.93
Schaftoside	2.60	1.84	2.32	2.13	2.90	2.81	5.90	102.11	2.93
Isoviolanthin	1.38	1.37	1.65	1.45	39.37	39.44	78.95	100.81	1.16
Rutin	1.50	2.57	2.45	2.190	6.61	6.32	12.91	100.72	1.84

### Sample quantitative analysis

**Fig. 4:**
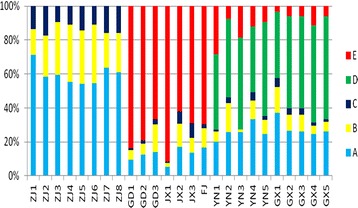
Content percentage of 5 respectively compounds in *Dendrobium officinale*, including Apigenin-6,8-di-C-β-d-glucoside (A), Apigenin-6-C-β-d-xyloside-8-c-β-d-glucoside (B), Schaftoside (C), Isoviolanthin (D), Rutin (E)

There are several components in *D. officinale* that were identified, such as apigenin-6,8-di-C-β-d-glucoside, isoschaftoside, schaftoside, violanthin, isoviolanthin, rutin, apigenin-6-C-β-d-xyloside-8-C-β-d-glucoside, and apigenin-6-C-β-d-glucoside-8-C-β-d-xyloside. Among these components, the contents of the 5 representative compounds were determined. The content analysis was performed to observe batch-to-batch variations for samples collected from different regions, as shown in Fig. 4. Although some differences exist in the composition of samples from different producing regions, the 25 batches samples could be divided into 3 categories. The results are consistent with our previous research that the production locations of *D. officinale* could be divided into three regions [22]. Apigenin-6-C-β-d-xyloside-8-C-β-d-glucoside, apigenin-6,8-di-C-β-d-glucoside and schaftoside are the common components that were found in all batches, whereas isoviolanthin and rutin are two marker ingredients that can be used to distinguish the source of this tonic medicine. *D. officinale* from Zhejiang province does not contain violanthin or rutin; however, the content of apigenin-6,8-di-C-β-d-glucoside is particularly high. Additionally, *D. officinale* from the Danxia landform region (Guangdong, Jiangxi, Fujian) has a high concentration of rutin but does not contain violanthin. In particularly, *D. officinale* growing in Guangxi and Yunnan contains violanthin. Because Yunan and Guangxi are geographically close to each other, the climate and environment of these two locations are similar, which may explain the reason why the compositions of the herb from these two provinces were almost identical. There are typical Danxia landform regions in Guangdong, Fujian and Jiangxi provinces, and the ultraviolet radiation is more intense. Consequently, the concentration of rutin is higher. The Zhejiang native species is of special provenance, and the chemical composition is different from those with other origins.

The validated method was successfully applied to determine the 5 representative compounds in the 25 batches of *D. officinale*, and the results are shown in Table 4. The average total contents of apigenin-6,8-di-C-β-d-glucoside (71.04 μg/g) in the samples from Zhejiang were the highest, followed by those for the Danxia landform area (Fujian, Guangdong and Jiangxi) (61.42 μg/g) and the Guangnan area (Guangxi and Yunan) (59.84 μg/g). Among the 5 detected compounds, the concentration of rutin in the herbs from the Danxia landform area was the highest (Fujian, Guangdong and Jiangxi), with an average content of 375.87 μg/g. The average content of rutin in the samples from the Guangnan area was 29.30 μg/g. Rutin was not present in the Zhejiang native species. Isoviolanthin could be detected only in the samples from the Guangnan, area with an average content of 112.89 μg/g. In contrast, schaftoside was detected in the herbs from all regions. The mean content in the Zhejiang native species was 15.64 μg/g, the mean content in the Danxia landform area species was 14.93 μg/g, and the mean content in the Guangnan area species was 5.50 μg/g. Meanwhile, the concentration of apigenin-6-C-β-d-xyloside-8-C-β-d-glucoside in the Zhejiang native species was 31.50 μg/g, the concentration in the Danxia landform area species was 46.79 μg/g, and the concentration in the Guangnan area species was 18.98 μg/g.

**Table 4:** Contents [mean ± SD (n = 3)] of 5 flavonoids in samples 1–25 (μg/g)

Samples	Apigenin-6,8-di-C-β-d-glucoside	Apigenin-6-C-β-d-xyloside-8-C-β-d-glucoside	Schaftoside	Isoviolanthin	Rutin
GD1	68.42 ± 0.43	41.04 ± 0.71	9.04 ± 0.15	ND	606.82 ± 0.24
GD2	70.07 ± 0.49	36.33 ± 0.29	11.37 ± 0.13	ND	447.91 ± 1.42
GD3	112.62 ± 0.24	19.82 ± 0.37	23.47 ± 0.26	ND	530.42 ± 0.42
JX1	20.62 ± 0.23	10.01 ± 0.15	2.31 ± 0.03	ND	364.25 ± 2.33
JX2	42.51 ± 0.12	34.66 ± 0.04	18.41 ± 0.08	ND	155.22 ± 0.11
JX3	48.36 ± 0.22	29.75 ± 0.18	30.82 ± 0.12	ND	243.81 ± 0.39
FJ	67.64 ± 0.15	46.74 ± 0.33	9.14 ± 0.14	ND	282.28 ± 0.53
ZJ1	86.63 ± 0.47	18.48 ± 0.32	16.61 ± 0.32	ND	ND
ZJ2	58.82 ± 0.43	24.31 ± 0.47	17.77 ± 0.32	ND	ND
ZJ3	105.55 ± 0.68	54.84 ± 0.15	16.73 ± 0.31	ND	ND
ZJ4	68.86 ± 1.28	42.24 ± 0.84	13.60 ± 0.39	ND	ND
ZJ5	38.84 ± 0.81	22.46 ± 0.45	10.43 ± 0.09	ND	ND
ZJ6	61.91 ± 0.36	39.11 ± 0.55	12.65 ± 0.04	ND	ND
ZJ7	85.22 ± 0.81	27.38 ± 0.80	21.21 ± 0.54	ND	ND
ZJ8	62.74 ± 0.20	23.51 ± 0.16	16.30 ± 0.47	ND	ND
YN1	75.34 ± 0.44	21.60 ± 0.11	4.60 ± 0.13	164.74 ± 1.60	10.61 ± 0.60
YN2	84.70 ± 0.37	56.32 ± 0.11	11.43 ± 0.19	152.96 ± 0.29	24.81 ± 0.15
YN3	111.30 ± 0.31	52.54 ± 0.27	1.20 ± 0.11	232.73 ± 0.34	79.82 ± 0.21
YN4	36.34 ± 0.29	12.52 ± 0.27	5.62 ± 0.11	42.61 ± 0.31	13.31 ± 0.21
YN5	34.73 ± 0.41	12.08 ± 0.31	2.91 ± 0.32	78.83 ± 0.47	13.87 ± 0.14
GX1	90.51 ± 0.37	37.21 ± 0.12	12.20 ± 0.09	94.89 ± 0.74	8.89 ± 0.81
GX2	37.10 ± 0.31	13.53 ± 0.23	5.30 ± 0.09	76.77 ± 0.48	8.31 ± 0.17
GX3	37.04 ± 0.29	13.90 ± 0.06	5.31 ± 0.15	76.98 ± 0.82	8.34 ± 0.26
GX4	43.51 ± 0.17	8.52 ± 0.27	3.32 ± 0.16	100.25 ± 0.16	20.12 ± 0.15
GX5	47.98 ± 0.28	9.96 ± 0.24	2.91 ± 0.12	110.87 ± 0.60	10.94 ± 0.27

Guangdong provinces (No. GD1–GD3), Jiangxi provinces (No. JX1–JX3), Fujian provinces (No. FJ), Zhejiang (No. ZJ1–ZJ8), Yunnan provinces (No. YN1–YN5), Guangxi provinces (No. GX1–GX5)

*ND* not detected

### HCA analysis

**Fig. 5:**
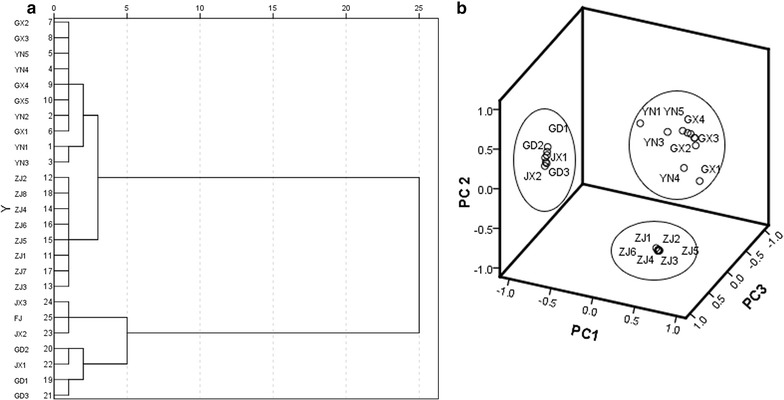
Dendrogram of hierarchical cluster analysis (HCA) for 25 samples of *Dendrobium officinale* (**a**), Principal component analysis (PCA) for 25 samples of *Dendrobium officinale* (**b**)

To investigate the similarities in *D. officinale* from different sources, we collected 25 batches of samples with 5 representative components, and a cluster analysis was performed. The results are shown in Fig. 5a. The cluster result is consistent with the contents of the components in the different samples. These samples can be divided into 3 categories. *D. officinale* samples from Zhejiang province, which did not contain rutin or isoviolanthin, were ground together. Samples from Guangxi and Yunnan provinces were ground together because they both contained isoviolanthin, and samples from Fujian, Guangdong and Jiangxi provinces had a high content of rutin and were ground together. The cluster results agreed with prior studies regarding the major producing regions of *D. officinale*. The presence of isoviolanthin in the samples only from the Yunnan and Guangxi provinces was also confirmed by the results of another research team, which suggests that the sources of our samples were reliable.

### PCA analysis

After importing all data into the SPSS 23.0 software to perform multivariate statistical analysis, the variance contribution rates of the difference components were provided. The variance contribution rate of component 1 was 64.671%, whereas for component 2, it was 19.990%. The variance contribution rate of component 3 was 12.932%. The cumulative variance contribution rate of these three components was 97.593%. The principal component spatial distribution map of the 25 batches of *D. officinale* is shown in Fig. 5b. The samples were ground into three different categories in three-dimensional space. The results were similar to those of the HCA analysis. It is feasible to apply these two methods to process the experimental data to objectively determine the differences in this medicinal material from different producing regions. These methods could be potentially developed to identify *D. officinale* from different producing regions.

## Conclusion

Good standard and good quality are two critical factors for TCM internationalization. The quality of material medicine resources has had a considerable impact on the development of the health industry, which has created a bottleneck for TCMs and has drawn widespread attention. Thus far, the Chinese Pharmacopoeia still uses the contents of mannose as a quality control index of *D. officinale*, which is lacking in specificity. *D. officinale* is planted in many places in China; however, the Chinese Pharmacopoeia clearly only considers *D. officinale* from one production location as a reference medicine.

In this study, we investigated the chromatographic fingerprint and quantitative analysis of component markers for quality control of *D. officinale*. HCA was used to analyze the samples from different areas, and all samples from the different regions could be grouped into 3 classes. In contrast to another research groups who have used the leaf, which is not the medicinal part of the plant, as the object of their studies or samples from only one location, we collected a large number of *D. officinale* samples from the main producing region in China. The results suggest that there are certain specific flavonoids in samples from different regions of production. We discovered that the contents of apigenin-6,8-di-C-β-d-glucoside, etc. (five representative substances in samples from different locations) exhibited significant differences.

We advise that *D. officinale* from traditional producing locations, such as Zhejiang provinces or the Danxia landform area, should be added as standard medicinal references. Additionally, the chromatographic fingerprint combined with quantification could be applied to distinguish and provide quality control for *D. officinale* samples from different regions of production, which can provide certain references for the Chinese Pharmacopoeia Commission’s revised Chinese Pharmacopoeia of quality standards for *D. officinale*.

## Abbreviations

*D. officinale*: *Dendrobium officinale*
TCM: Traditional Chinese Medicine
HPLC–ESI–MS/MS: high performance liquid chromatography-electrospray ionization/mass spectrometry
HCA: hierarchical cluster analysis
t_R_: retention times
